# Wernicke's Encephalopathy Following Semaglutide Treatment for Obesity: A Systematic PRISMA Review of Case‐Based Evidence

**DOI:** 10.1002/oby.70256

**Published:** 2026-07-03

**Authors:** Janice Bidesie, Erik Oudman

**Affiliations:** ^1^ Experimental Psychology Helmholtz Institute, Utrecht University Utrecht the Netherlands; ^2^ Slingedael Center of Expertise for Korsakoff Syndrome Lelie Care Group Rotterdam the Netherlands; ^3^ Long Term Intensive Treatment Parnassia Psychiatric Institute The Hague the Netherlands; ^4^ Department of Public Health and Primary Care Leiden University Medical Center Leiden the Netherlands

**Keywords:** gastrointestinal adverse effects, GLP‐1 receptor agonists, nutritional deficiency, obesity, pharmacovigilance, semaglutide, thiamine deficiency, Wernicke's encephalopathy

## Abstract

**Objective:**

Glucagon‐like peptide‐1 receptor agonists are increasingly prescribed for obesity. Although generally considered safe, marked appetite suppression and persistent gastrointestinal adverse effects may increase the risk of nutritional deficiencies. Wernicke's encephalopathy (WE), caused by thiamine deficiency, is a rare but potentially fatal neurological disorder that may result in irreversible cognitive impairment if not promptly recognized and treated. Recently, cases of WE associated with semaglutide use have been reported. This study aimed to systematically review published case reports of WE occurring after semaglutide treatment for obesity from an endocrinological drug safety perspective.

**Methods:**

A systematic literature search was conducted in PubMed, Embase, CINAHL, and Scopus according to PRISMA guidelines. Case reports were included when semaglutide was prescribed for obesity and the presentation was consistent with WE.

**Results:**

Six cases were identified. All patients experienced prolonged gastrointestinal symptoms and substantial weight loss before neurological deterioration. Common manifestations included altered mental status and oculomotor abnormalities. Outcomes were poor in several cases, including progression to Korsakoff syndrome or death.

**Conclusions:**

Clinicians should remain vigilant for thiamine deficiency in patients with persistent gastrointestinal intolerance or rapid weight loss during GLP‐1 treatment, as early parenteral thiamine administration is essential to prevent irreversible neurological sequelae.

## 1 Introduction

Obesity is a rapidly growing global health concern, with more than 1 billion people worldwide now classified as living with the condition [1]. Its prevalence has risen sharply across all age groups and socioeconomic strata, and obesity is a major risk factor for type 2 diabetes, cardiovascular disease, musculoskeletal disorders, certain cancers, and premature mortality [2, 3]. Although lifestyle interventions remain the cornerstone of treatment, long‐term weight maintenance is often unsuccessful, while bariatric surgery, despite its effectiveness, is invasive and associated with surgical risks and long‐term nutritional complications [4, 5, 6]. The urgent need for effective, safe, and sustainable treatment options has driven a paradigm shift in obesity care. Consequently, there is a pressing need for effective, scalable, and safe pharmacological therapies for obesity.

Glucagon‐like peptide‐1 receptor agonists (GLP‐1 RAs), particularly semaglutide (i.e., Ozempic, Wegovy), have revolutionized the management of obesity. Large randomized controlled trials have shown that once‐weekly semaglutide produces weight loss of up to 15% of baseline body weight, while also improving glycemic control, cardiovascular outcomes, and health‐related quality of life [7, 8, 9]. Its efficacy has been shown to rival bariatric surgery in some populations, leading to widespread uptake in both diabetic and non‐diabetic individuals [10]. Semaglutide induces weight loss primarily through central appetite suppression and delayed gastric emptying, leading to reduced caloric intake and early satiety [7]. In the absence of adequate nutritional compensation, prolonged nausea, vomiting, or anorexia may precipitate micronutrient deficiencies. In most patients these effects are transient and clinically manageable; however, in susceptible individuals they may result in clinically significant nutritional compromise. Thiamine stores may become critically depleted within weeks under conditions of reduced intake or increased metabolic demand [11, 12, 13, 14]. During periods of rapid weight loss, cerebral energy metabolism becomes increasingly dependent on thiamine‐dependent enzymatic pathways, including pyruvate dehydrogenase and α‐ketoglutarate dehydrogenase. Similar pathophysiological mechanisms have been well described in bariatric surgery–related Wernicke's encephalopathy (WE) and in hyperemesis gravidarum, supporting the biological plausibility of thiamine deficiency–related neurological complications in patients treated with GLP‐1 RAs [6, 12, 13, 15, 16].

Wernicke's encephalopathy is an acute, life‐threatening neuropsychiatric syndrome caused by thiamine deficiency. First described by Carl Wernicke in 1881, it remains preventable yet underdiagnosed, with high morbidity and mortality [12]. While traditionally linked to alcohol use disorder, WE is increasingly recognized in a range of non‐alcoholic conditions, including hyperemesis gravidarum, malignancy, bariatric surgery, anorexia nervosa, inflammatory bowel disease, and chronic kidney disease [12, 13, 14, 15, 16]. The clinical diagnosis is challenging, as the classic triad of confusion, ocular abnormalities, and ataxia is observed in only one‐third of patients [13]. Autopsy studies confirm that the majority of cases remain undiagnosed during life [17, 18]. Prompt, high‐dose parenteral thiamine is required for effective treatment, as oral supplementation is insufficient in acute deficiency states. Delayed or inadequate dosing increases the risk of progression to chronic Wernicke–Korsakoff syndrome, characterized by chronic amnesia and confabulation [14].

Although large‐scale clinical trials of semaglutide have not reported WE as an adverse outcome, emerging case reports describe WE in patients treated with semaglutide for obesity. These reports raise concern that prolonged gastrointestinal side effects and rapid weight loss associated with GLP‐1 RA therapy may precipitate thiamine deficiency and severe neurological complications in susceptible individuals. The aim of this systematic review is therefore to synthesize the available case‐based evidence on WE occurring following semaglutide treatment for obesity, with specific attention to clinical risk factors and implications for endocrine practice.

## 2 Methods

We conducted a systematic review of the literature and included only reports that provided sufficient information on individual patients. This systematic review was conducted in accordance with the Preferred Reporting Items for Systematic Reviews and Meta‐Analyses (PRISMA 2020) guidelines. The protocol was prospectively registered in PROSPERO (ID 1299720). Eligibility criteria were:

2.1

- Population: Adults treated with semaglutide for obesity.
- Exposure: Semaglutide (Ozempic or Wegovy).
- Outcome: Clinically diagnosed WE.
- Study design: Case reports or case series with individual‐level data.

Accepted methods of diagnosing WE were: Caine's operational criteria [19], the classic triad of confusion and/or ataxia and/or ocular abnormalities and/or autopsy confirmation, or a documented clinical response to parenteral thiamine. Case studies published in any language were eligible for inclusion.

A systematic search was performed in MEDLINE, Embase, CINAHL, and Scopus from inception until February 10, 2026. The search strategy combined the terms: “semaglutide,” “Ozempic,” “Wegovy,” “GLP‐1 receptor agonist,” “Wernicke encephalopathy,” and “Korsakoff syndrome.” Titles, abstracts, and full texts were screened independently by two reviewers. Discrepancies were resolved by consensus. Reference lists of included articles were also screened for additional reports.

Data were extracted from eligible publications using a structured template. The following variables were collected: year of publication, patient age, sex, indication for semaglutide, treatment duration and dosing, presenting signs of WE, presence of ataxia, ocular motor abnormalities and mental status changes, neuroimaging findings (MRI/CT), thiamine treatment regimen, and clinical outcome. Suboptimal treatment of WE was defined as < 500 mg of parenteral thiamine as the initial dose. Reports were excluded if insufficient clinical information was available to confirm WE or if no patient‐level details were provided.

Descriptive analyses were performed using SPSS (version 25.0). We calculated medians, ranges, standard deviations (SD), frequencies, and percentages for demographic data, clinical features of WE, treatment characteristics, and patient outcomes. The number of cases available for each specific analysis is indicated in brackets throughout the results.

Diagnostic evaluation and exclusion of alternative causes of encephalopathy varied across the included case reports. Information on alcohol use, prior bariatric surgery, and other potential risk factors for thiamine deficiency was not consistently reported. Likewise, the extent to which alternative diagnoses such as delirium, metabolic encephalopathy, cerebrovascular events, or medication‐induced encephalopathy were systematically excluded differed between reports.

## 3 Results

**FIGURE 1:**
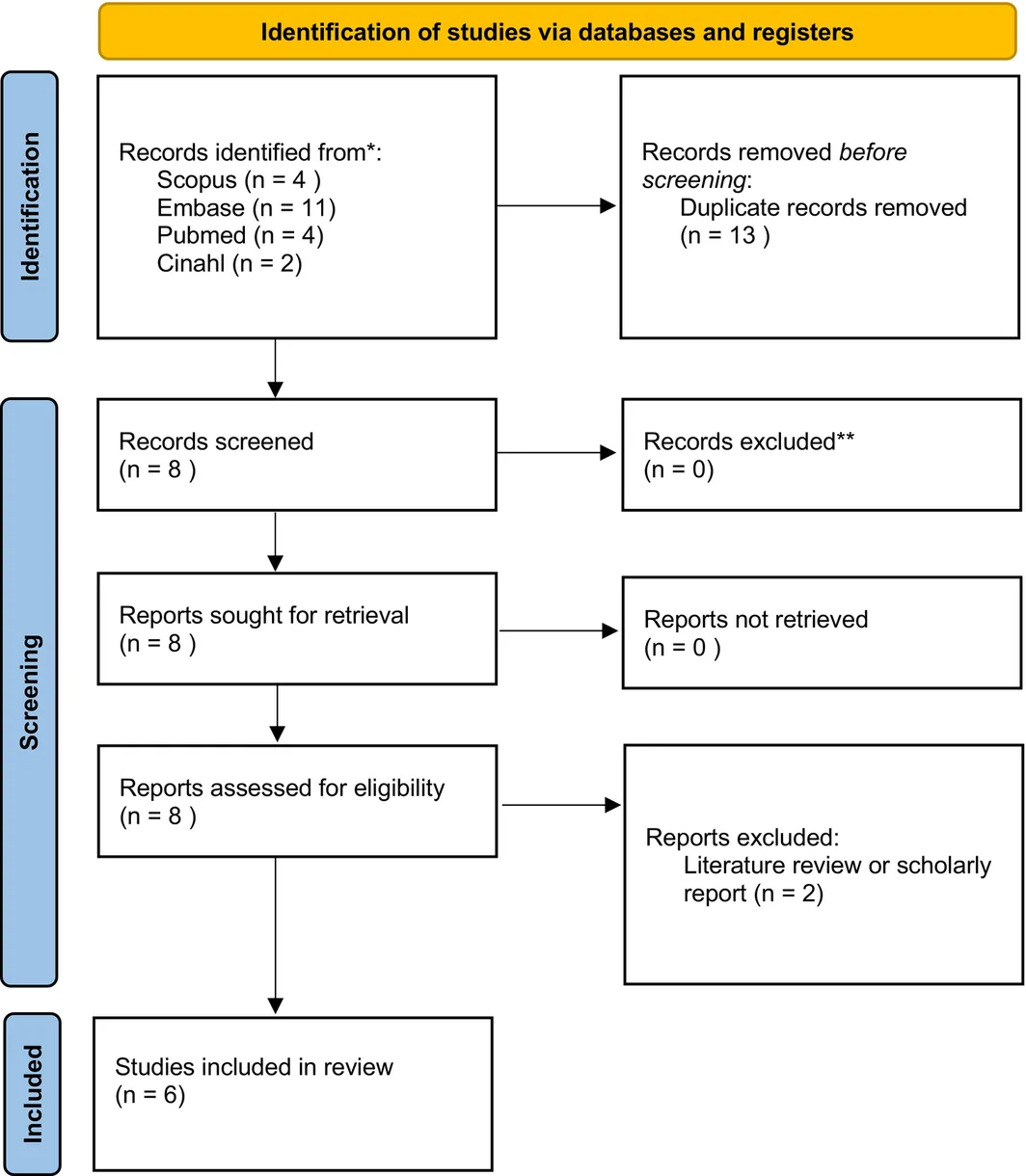
PRISMA flow diagram.

We performed our search on February 10, 2026. A flowchart is presented in Figure 1. All included studies were case reports because group studies were not available, since information on the course of illness and symptomatology was lacking in all group studies. After full text screening, we identified six unique cases diagnosed with WE in patients who were prescribed Ozempic or Wegovy for weight loss in obesity [20, 21, 22, 23, 24, 25].

### 3.1 Patient Characteristics

WE was reported in four female and two male patients who were prescribed semaglutide for obesity. The mean age was 47.2 (SD: 13.9, range: 37–74 years), and patients had an average duration of semaglutide treatment of 4.9 months (SD: 3.6 months, range: 2.5–12 months). Importantly, five out of six cases had comorbid somatic and/or psychiatric disorders such as anxiety, COPD, diabetes mellitus, hyperlipidemia, hypertension, major depressive disorder, polysubstance abuse, and thyroid disease [20], hypertension and obstructive sleep apnea [21], ischemic stroke, coronary artery disease, hypertension, hypothyroidism, depression, and diabetes mellitus type 2 [22], Cushing's syndrome [24], and chronic kidney disease, hypothyroidism, and diabetes mellitus [25]. Laboratory assessment of thiamine levels and nutritional status was inconsistently reported across the included case studies.

### 3.2 Presenting Signs of WE After Semaglutide Treatment

Loss of appetite (five out of six cases) [20, 21, 22, 24, 25], weakness (four out of six cases) [20, 22, 23, 25], vomiting (three out of six cases) [20, 22, 25], constipation (two out of five cases) [22, 23], other gastrointestinal issues (one case) [21], and asthenia (one case) [24] were reported before onset of WE (six cases) [20, 21, 22, 23, 24, 25].

### 3.3 WE: Classic Triad

WE is characterized by the classic triad of ataxia, eye movement disorders, and mental status change [13, 26]. The most frequently observed characteristic of the classic WE triad in the reviewed cases was mental status change (confusion/delirium or memory loss), which was present in five out of six cases [21, 22, 23, 24, 25]. Moreover, five out of six case studies also showed signs of nystagmus of the eyes [20, 21, 22, 24, 25]. Finally, four out of six cases showed signs of ataxic gait disturbance [21, 23, 24, 25]. The full triad was present in three out of six cases [21, 24, 25].

### 3.4 Imaging

Enhanced signal intensity on MRI in the mammillary bodies, medial thalamus, and the periaqueductal gray matter is associated with WE. On CT hypoattenuations in the same regions can be visible [13, 26]. Of the six cases, five underwent MRI [20, 21, 23, 24, 25], one patient underwent EEG [21], and one patient underwent CT [20]. Of the cases that underwent MRI, all showed classical signs of WE. The most diffuse pattern was seen in the first and last study, showing additional involvement of the basal ganglia and brainstem [20] or the corpus callosum and pons [25]. In the second case study MRI alterations were restricted to the mammillary bodies [21]. Alterations were also visible on EEG, showing diffuse background slowing [21]. The CT finding did not show any signs [21].

### 3.5 Treatment and Outcome

For three cases, the treatment regimen was fully explained [20, 21, 24]. In two cases, the patient was started on 500 mg thiamine IV and later switched to 3 × 500 mg thiamine IV [20, 21]. In the last case study, 3 × 500 mg thiamine IV was immediately started [24]. Regarding follow‐up, one patient died following WE [22]. One patient had a good outcome [20]. The remaining four cases showed prolonged memory issues related to chronic Korsakoff syndrome [21, 23, 24, 25]. Of importance, the last case study also resulted in chronic loss of leg function [25].

## 4 Discussion

This systematic review provides a synthesis of published case reports describing WE associated with the use of semaglutide for obesity. Although six cases were identified, several consistent patterns emerge. Patients were typically middle‐aged adults, often with multimorbidity, who developed gastrointestinal side effects such as nausea, vomiting, anorexia, and constipation prior to neurological deterioration. In nearly all reports, the cardinal features of WE, particularly mental status changes and oculomotor abnormalities, were present, and MRI findings were consistent with thiamine deficiency. Outcomes were variable: while one patient recovered fully, four developed persistent cognitive deficits consistent with Korsakoff syndrome, and one patient died.

Importantly, these observations align with a growing body of literature demonstrating that WE is not confined to alcohol misuse but represents a final common pathway of thiamine deficiency across diverse medical conditions, including bariatric surgery, hyperemesis gravidarum, inflammatory bowel disease, malignancy, and chronic kidney disease [12, 13, 14, 15, 16]. In these settings, a combination of reduced intake, impaired absorption, and increased metabolic demand leads to rapid depletion of thiamine stores, which are limited and may be exhausted within weeks [13, 14]. The present cases suggest that semaglutide‐associated WE may arise through analogous mechanisms. Importantly, in the six cases presented here, the development of thiamine deficiency was likely accelerated by the presence of multiple comorbidities and additional clinical risk factors.

The management of thiamine deficiency has been extensively studied. Oral thiamine is inadequate in acute deficiency states because intestinal absorption is limited and unreliable [14]. Instead, high‐dose parenteral thiamine is required: recommended regimens are ≥ 500 mg intravenously three times daily for suspected or confirmed WE [14, 26]. Delayed or insufficient therapy substantially increases the risk of progression to Korsakoff syndrome, characterized by irreversible memory impairment and confabulation [14, 26]. In this review, several patients received suboptimal thiamine dosing and subsequently developed persistent cognitive deficits or died, echoing prior reports that inadequate early treatment predicts poor outcomes [14, 26].

Clinically, these findings underscore the need for vigilance in patients receiving GLP‐1 RAs. Persistent nausea, vomiting, or reduced intake should prompt early consideration of thiamine deficiency, ideally before neurological manifestations emerge. Prophylactic supplementation may be warranted in high‐risk individuals, though prospective studies are needed to evaluate its effectiveness. When WE is suspected, high‐dose parenteral thiamine must be administered immediately, without awaiting laboratory confirmation, as serum thiamine levels are neither sensitive nor specific for deficiency [13, 14].

Future research should address the incidence and risk factors for thiamine deficiency in semaglutide‐treated populations through pharmacovigilance programs and registry studies. Large‐scale cohorts could also assess whether preventive supplementation strategies are effective in mitigating risk. Until such data are available, clinicians should balance the well established cardiometabolic benefits of semaglutide [7, 8, 9, 10, 11] against the potential for rare but severe neurological complications, maintaining a high index of suspicion in symptomatic patients.

Recent pharmacovigilance data have identified multiple cases of WE associated with GLP‐1 RAs other than semaglutide, including tirzepatide [27]. In addition, retatrutide may also carry a risk of inducing thiamine deficiency due to its potent weight‐reducing effects. Rapid weight loss, reduced oral intake, nausea, and vomiting are considered important contributing factors to thiamine deficiency and subsequent neurological complications [27]. These findings suggest that the risk of thiamine deficiency may extend beyond semaglutide to other medications associated with rapid weight loss.

Despite these consistent clinical features, the available evidence has important limitations. All data are derived from case reports, which are prone to reporting bias and incomplete clinical documentation. The true incidence of semaglutide‐related WE is therefore unknown and may be underestimated, especially as up to 80% of WE cases remain undiagnosed during life [17, 18]. Furthermore, comorbidities and concomitant medications could have contributed to nutritional deficiency, complicating causal attribution. Finally, the primary and secondary indications of GLP‐1 treatment were not always strictly separated, suggesting that GLP‐1 in patients diagnosed with diabetes mellitus could also lead to WE.

Clinically, the reviewed cases highlight several important challenges. Early symptoms of thiamine deficiency are nonspecific and may overlap with expected adverse effects of GLP‐1 RA therapy, including fatigue, weakness, anorexia, and gastrointestinal discomfort. Reliance on the classic triad of confusion, ataxia, and ocular abnormalities is unsafe, as it is present in only a minority of patients [13]. Furthermore, up to 80% of WE cases remain undiagnosed during life, even in hospital settings [17, 18]. These diagnostic pitfalls are likely amplified in non‐alcoholic populations, where clinical suspicion for WE may be lower.

Persistent vomiting, severe appetite suppression, or rapid weight loss during semaglutide therapy should prompt early consideration of thiamine deficiency, particularly in patients with multimorbidity or prior nutritional vulnerability. Low‐threshold thiamine supplementation may be reasonable in high‐risk individuals, extrapolating from bariatric and hyperemesis populations, although prospective data are needed to define optimal preventive strategies [6, 12]. When neurological symptoms emerge, immediate administration of high‐dose intravenous thiamine is essential, regardless of alcohol history or laboratory results.

In conclusion, WE appears to be a rare but potentially devastating complication associated with semaglutide treatment for obesity, likely mediated by prolonged gastrointestinal intolerance, reduced nutritional intake, and rapid weight loss leading to thiamine deficiency. Outcomes are frequently poor when diagnosis or treatment is delayed. As the use of GLP‐1 RAs continues to expand, clinicians should maintain a high index of suspicion for WE in symptomatic patients and initiate prompt parenteral thiamine therapy when indicated. Further prospective studies are needed to clarify incidence, identify high‐risk populations, and develop evidence‐based prevention strategies, but until such data are available, heightened clinical vigilance is essential.

## Funding

The authors have nothing to report.

## Conflicts of Interest

The authors declare no conflicts of interest.

## Data Availability

The data that support the findings of this study are available on request from the corresponding author. The data are not publicly available due to privacy or ethical restrictions.
